# Microbial community analysis and biodeterioration of waterlogged archaeological wood from the Nanhai No. 1 shipwreck during storage

**DOI:** 10.1038/s41598-018-25484-8

**Published:** 2018-05-08

**Authors:** Zijun Liu, Tongtong Fu, Cuiting Hu, Dawa Shen, Nicola Macchioni, Lorena Sozzi, Yue Chen, Jie Liu, Xingling Tian, Qinya Ge, Zhengteng Feng, Huiru Liu, Zhiguo Zhang, Jiao Pan

**Affiliations:** 1 Ministry of Education Key Laboratory of Molecular Microbiology and Technology, Department of Microbiology, College of Life Sciences, Nankai University, Tianjin, 300071 P.R. China; 2 Chinese Academy of Cultural Heritage, Beijing, 100029 P.R. China; 3 CNR-IVALSA, Via Madonna del Piano 10, 1-50019 Sesto Fiorentino, Italy; 4 Maritime Silk Road Museum, Yangjiang, Guangdong province 529500 P.R. China; 5 National Center of Underwater Cultural Heritage, Beijing, 100192 P.R. China

## Abstract

Wooden shipwrecks are a significant part of the underwater cultural heritage. In 2007, the Nanhai No. 1 shipwreck was salvaged from the seabed and moved into the Marine Silk Road Museum, where it is still stored in a water tank. We analysed the microbial communities colonizing the hull surface of the Nanhai No. 1 shipwreck during storage. Six samples exposed to air were collected from different spots of the ship that exhibited obvious microbial plaques. High-throughput sequencing revealed the bacterial community includes both aquatic and terrestrial species, while in the fungal community, *Fusarium* was the most abundant genus across all samples and accounted for 84.91% to 98.40% of the total community composition. Two *Fusarium* species were isolated from the samples and were identified as *F*. *solani* and *F*. *oxysporum*. Both of the isolates were able to degrade cellulose, but only *F*. *solani* had the ability to degrade lignin. Antimicrobial efficacy in inhibiting the growth of *Fusarium* was assessed with five kinds of biocides, and isothiazolinones exhibited specific inhibition of *Fusarium* growth. These results provide critical background information to protect and reduce the biodegradation and destruction of this important historical shipwreck, and inform efforts to protect other similar artifacts.

## Introduction

The Nanhai No. 1 shipwreck is located in the city of Yangjiang in Guangdong Province which lies on the Chinese southern coast. The ship sank off the coast during the Southern Song Dynasty (1127–1279 AD) and was discovered in the summer of 1987. In 2007, several authorities cooperated to salvage the whole shipwreck from the seabed that was over 20 metres underwater (Supplementary Fig. 1) and moved it to the Marine Silk Road Museum, where it was stored in a tank with seawater. The successful recovery is an unprecedented achievement and a historical landmark for Chinese underwater archaeology. A full-scale excavation of the Nanhai No. 1 shipwreck began on land in November 2013. A number of archaeological materials including ornaments, wood combs, bronze mirrors and even plant seeds have been recovered from the shipwreck. Further excavation revealed that the coastal trader carried a cargo of gold, silver, and copper coins, in addition to a significant number of porcelain items for ocean-going trade^1^. The remains of the ancient vessel are expected to yield critical information on ancient Chinese shipbuilding and navigation technologies. As the most important underwater discovery in Chinese archaeological history, the 800-year-old shipwreck is of great significance to the history of overseas trade and is also an important resource for Maritime Silk Road research.

Over the past 100 years, several shipwrecks have been excavated, raised and conserved. For instance, the Viking-period Oseberg ship was found embedded in waterlogged clay at a land site excavation in Norway in 1902^2^. Additionally, the warship, *Vasa*, was raised in 1961 after 333 years in the brackish and cold waters of the Baltic Sea^3^. King Henry VIII’s warship *Mary Rose* was discovered in 1971 and raised in 1982^4^. Now in the final stages of conservation, it takes its place in a stunning and unique museum. However, another example is the Late Bronze Age Uluburun ship that sank off the Turkish coast at approximately 1335–1320 BC^5^. Under waterlogged and anaerobic conditions, archaeological wood is relatively well protected from biological decomposition compared to the degradation that occurs in most terrestrial environments. Some organisms, however, including bacteria and soft rot fungi, can still degrade waterlogged wood that is of significant archaeological value.

There are two main bacterial groups that degrade waterlogged wood: erosion bacteria and tunnelling bacteria. Experimental laboratory research and investigations of degraded archaeological wood from various terrestrial and aquatic sites worldwide have confirmed the presence of these two groups^6–8^. Erosion bacteria can degrade wood under very low oxygen concentrations, while tunnelling bacteria are widespread in nature, occurring in both terrestrial and aquatic environments and can tolerate a wide range of temperatures and humidity^9^. The more commonly reported bacteria associated with wood-decay environments are cellulolytic aerobes such as *Cytophaga*, *Cellvibrio*, anaerobic genera such as *Clostridium*, and cosmopolitan taxa such as *Bacillus*, *Pseudomonas*, *Arthrobacter*, *Flavobacterium*, and *Spirillum*^10,11^. Fungi that cause soft-rot usually belong to the *Ascomycota* and are able to degrade wood in terrestrial and aquatic environments, having low oxygen requirements. Fungal taxa associated with soft-rot wood decay in terrestrial environments, include among others, the genera *Cladosporium*, *Acremonium*, *Fusarium*, and *Chaetomium*^12^. In the marine environment, wood is degraded by marine fungal taxa that have been recorded since 1944 by Barghoorn and Linder and shown to have lignocellulolytic capacity under laboratory conditions as revealed by Gareth Jones since 1971^13,14^.

As the excavation progressed on land since November 2013, the upper deck of Nanhai No. 1 has been exposed to air, while the integral hull remained immersed in seawater. Non-woven fabrics were used to cover the areas directly exposed to air, and a spraying system was also used to maintain moisture. Meanwhile, borate buffer solution (BBS) was applied every week to prevent potential microbial contamination. However, in October 2014, fungal mycelia, in addition to salt precipitates, started to develop on some areas where the fabrics did not cover the wood appropriately. By using high-throughput sequencing techniques and culture-based methods, the aim of this study was to identify potential wood-degrading microorganisms that are responsible for the degradation of the Nanhai No. 1 shipwreck during storage. For this purpose, microbial community analysis was undertaken specifically on wood exposed to air. Furthermore, we investigated the enzymatic characteristics of the dominant fungi that were implicated in wood degradation. Finally, antimicrobial efficacy in inhibiting the growth of the dominant fungi was assessed with five kinds of biocides.

## Results

### Microscopic observation

**Figure 1:**
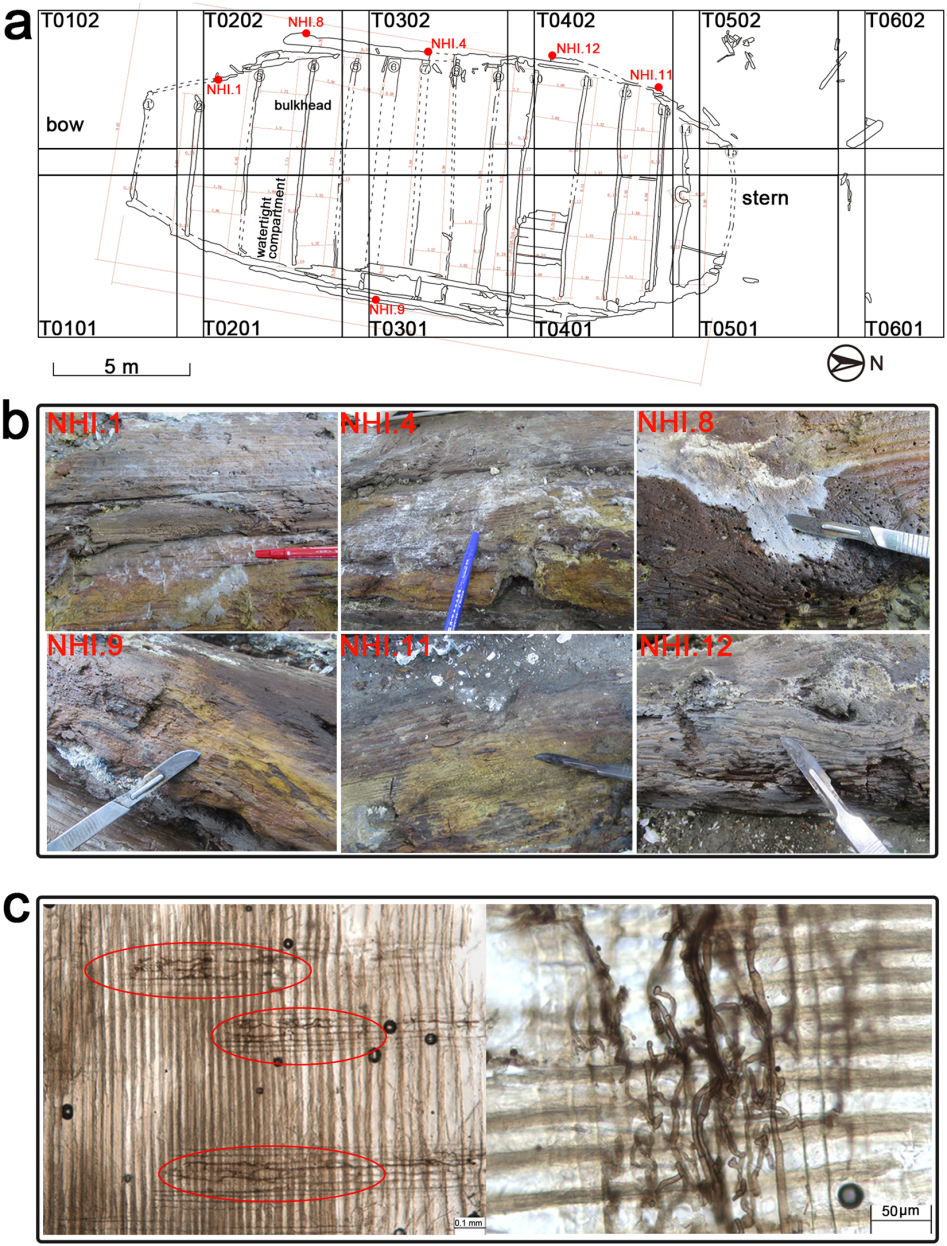
Specific locations of the sampling sites on the Nanhai No. 1 ship’s hull. (**a**) The platform of the shipwreck. T0101–T0602 are different excavation areas. We gratefully acknowledge Jian Sun from the National Center of Underwater Cultural Heritage for providing the image. (**b**) Images of six samples that were taken from the ship’s hull. Samples were taken by using sterile scalpels and placing them into 2 mL micro-centrifuge tube for subsequent microscopic observation, DNA extraction, and cultivation. (**c**) Transmission light microscope images showing fungal hyphae that had penetrated the wood structure. Red circles indicate fungal hyphae.

The detailed sampling spots and sample characteristics are shown in Fig. 1a–b. Samples were observed under a transmission light microscope and exhibited typical morphological characteristics of filamentous fungi (Fig. 1c).

### Microbial community analyses

**Figure 2:**
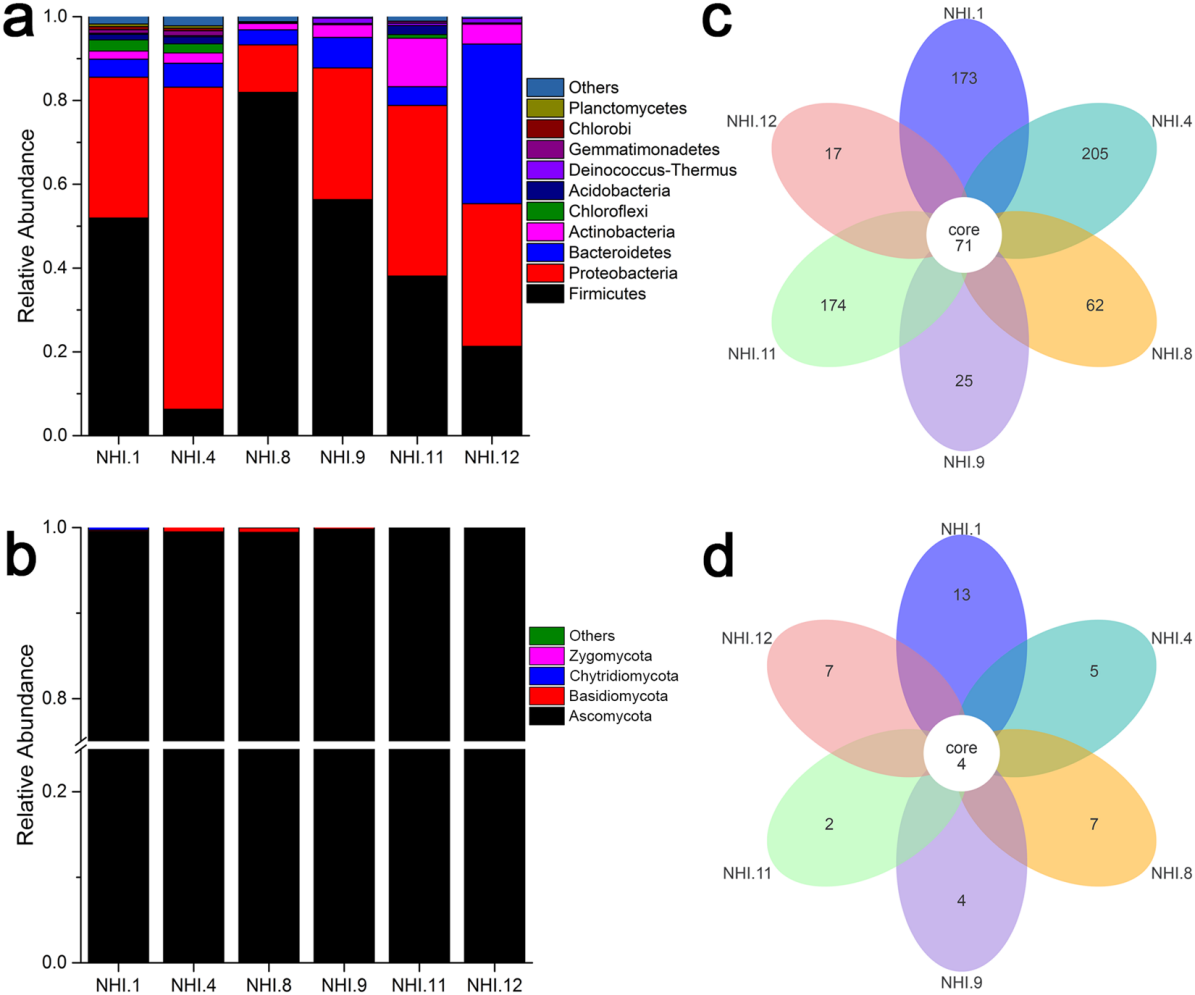
Relative abundance of the ten most abundant microbial phyla and Venn diagrams showing shared OTU diversity among the six samples. (**a**) Relative abundance for each sample is shown out of 100%. Bacterial phyla are coloured according to the legend on the right. (**b**) Relative abundance for each sample is shown out of 100%. Fungal phyla are coloured according to the legend on the right. (**c**) Bacterial OTUs and (**d**) fungal OTUs that were shared among samples.

Bacterial community composition (via 16S rRNA V4 region sequencing) was assessed by amplicon sequencing using the Illumina HiSeq2500 PE250 platform for the six samples. A total of 284,855 high-quality reads were obtained after filtering low-quality reads, chimaeras and trimming adapters, barcodes and primers. All 16S rRNA gene sequences were assigned to 43 bacterial phyla. The ten most abundant taxa at the phylum level are summarized in Fig. 2. Firmicutes were the most abundant phylum and represented between 6.30 and 82.92% of each sample’s reads with an average relative abundance of 42.65%. Proteobacteria were the second most abundant phylum with an average relative abundance of 38.00%. Bacteroidetes comprised 38.09% of all reads in sample NHI.12 but only 3.60 to 7.30% in the other five samples. Other major phyla consisted of the Actinobacteria (1.54–11.58%, average of 4.22%), Chloroflexi (0.13–2.66%, average of 1.04%) and Acidobacteria (0.02–2.13%, average of 0.90%) (Fig. 2a, Supplementary Table 1).

There was a total of 323,029 fungal community reads after filtering low-quality reads, chimaeras and trimming adapters, barcodes and primers. Four fungal phyla were present in the six samples (Fig. 2b). Ascomycota was the most dominant phylum, accounting for 99.45 to 99.98% of each sample’s reads, with an average relative abundance of 99.71%. The remaining phyla including the Basidiomycota, Chytridiomycota, and Zygomycota (Mucoromycota, Zoopagomycota) accounted for 0.29% of the remaining fungal reads. The genus-level relative abundance of the fungal communities is summarized in Table 1. Dominant fungal genera were similar among all samples. Among the ten most abundant fungal taxa, *Fusarium* and *Aspergillus* were present in all samples, and *Fusarium* was the most abundant genus across all samples, accounting for between 84.89% and 98.41% of the total community, with an average abundance of 93.53%. At the species level, *F*. *oxysporum* and *F*. *solani* were the dominant *Fusarium* species, and *F*. *solani* was the more abundant species of the two (Table 2).

**Table 1:** Relative abundance of dominant fungi among samples at the genus level.

Dominant Genus	NHI.1 (%)	NHI.4 (%)	NHI.8 (%)	NHI.9 (%)	NHI.11 (%)	NHI.12 (%)
*Fusarium*	96.89	97.07	92.35	98.40	84.91	91.58
*Candida*	0.66	0.38	0	0	0	0
*Aspergillus*	0.10	0.31	0.12	0.48	0.15	0.21
*Wallemia*	0	0.51	0	0	0	0
*Olpidiaster*	0.31%	0	0	0	0	0
*Meyerozyma*	0	0.26	0.02	0.02	0	<0.01
*Acremonium*	0.15	<0.01	0	<0.01	0	0
*Conocybe*	0.13	0	0	0	0	0
*Monascus*	0	0	0.12	0.04	0.11	0.08
*Monographella*	<0.01	0	0	0.06	0	0.10

**Table 2:** Relative abundance of dominant *Fusarium* species among samples.

Dominant Species	NHI.1 (%)	NHI.4 (%)	NHI.8 (%)	NHI.9 (%)	NHI.11 (%)	NHI.12 (%)
*Fusarium solani*	92.81	86.57	92.32	98.40	84.89	91.57
*Fusarium oxysporum*	4.08	10.50	<0.01	<0.01	<0.01	<0.01

Sample overlap was assessed to identify shared diversity among the six samples (Fig. 2c–d). A total of 71 bacterial operational taxonomic units (OTUs) were shared among samples (a total richness of 6,072 OTUs). Additionally, the six samples shared four fungal OTUs out of a total fungal richness of 280 OTUs. These results indicated that microbial communities overlapped to some extent among different sampling sites. This was particularly evident for fungal communities, in which *Fusarium* was the major genus and comprised a major proportion of all communities.

### Isolation and identification of dominant fungi

**Figure 3:**
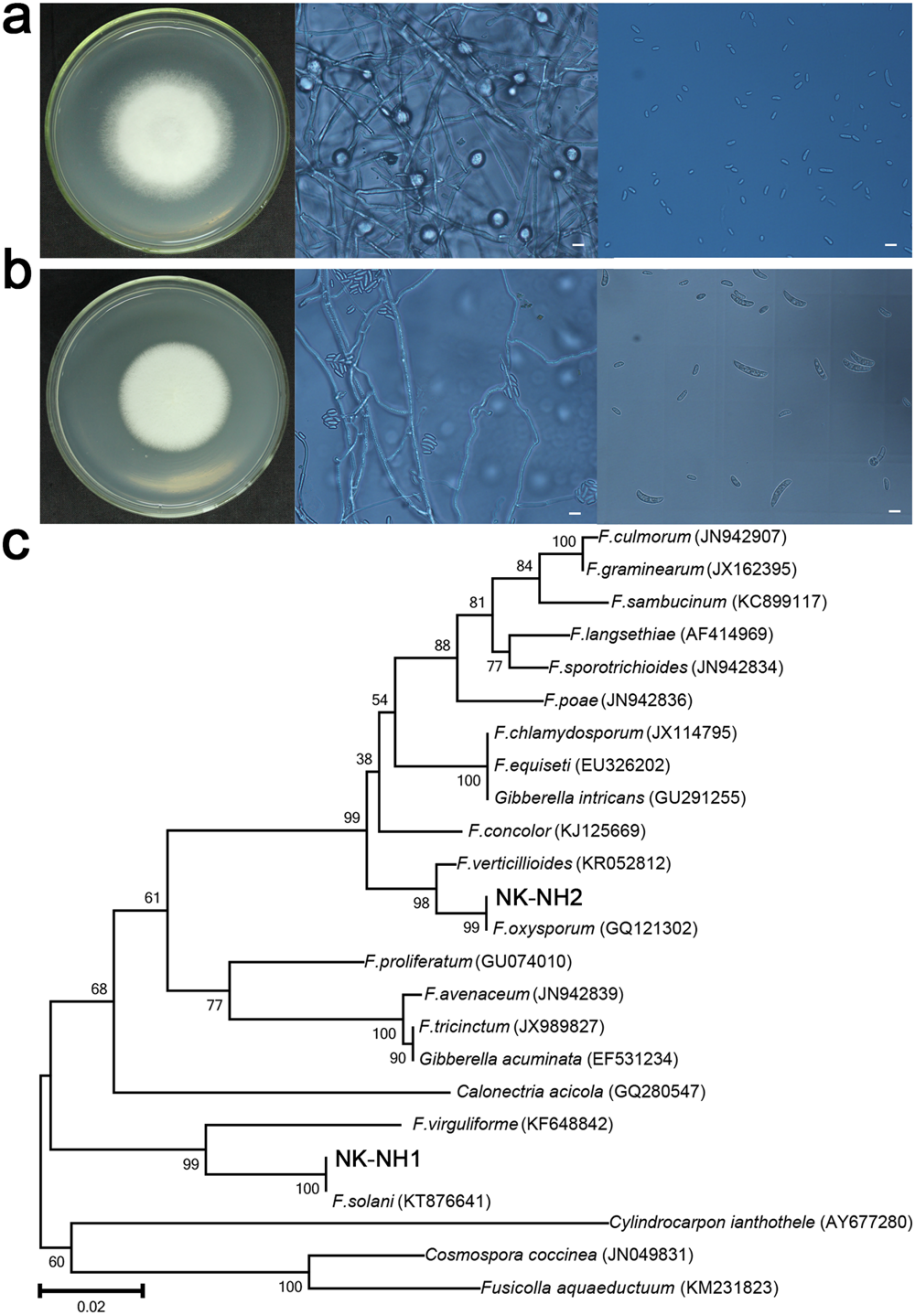
Colony and micro-morphology features of two fungal isolates at 400x magnification. (**a**) NK-NH1. Scale bar is 10 μm. (**b**) NK-NH2. Scale bar is 10 μm. (**c**) Neighbour-joining phylogenetic tree of *Fusarium* sp. NK-NH1 and *Fusarium* sp. NK-NH2 ITS gene sequences (~520–540 bp sequence used for each). Representatives of the most closely related strains and additional members of the genus *Fusarium* are included for taxonomic context. Bootstrap values at nodes are given as a percentage of 1000 bootstrap replicates. Scale bar indicates the expected number of substitutions/site.

The high-throughput sequencing results revealed a dominance of *Fusarium* among the six samples, and we therefore attempted to isolate the dominant fungi to study their enzymatic characteristics. Incubation on potato dextrose agar (PDA) plates at 28 °C for 6 days resulted in the presence of two colonies (Fig. 3a–b). Two strains, ‘NK-NH1’ and ‘NK-NH2’, were isolated from the samples. NK-NH1 grew rapidly with abundant aerial mycelia on PDA, and colonies sometimes exhibited a purple colour on the upper surface with some cream coloration in the centre of the colony. Microconidia were present, varying from sparse to abundant and were generally single-celled and oval to kidney-shaped. Macroconidia were abundant, stout, and generally cylindrical, with the dorsal and ventral surfaces parallel for most of their length. Chlamydospores were also observed for NK-NH1. NK-NH2’s growth was rapid with white aerial mycelia. Microconidia were abundant and generally single-celled, oval to kidney-shaped and were produced only in false heads. Macroconidia were abundant and only slightly sickle-shaped.

We then sequenced the 28S rRNA genes and the ITS1–5.8S rRNA-ITS2 gene regions for both colonies. NK-NH1 displayed 99% and 100% sequence similarity to the 28S rRNA gene and ITS sequences of F. solani, respectively. NHI.2 displayed 100% and 99% sequence similarity with 28S rRNA gene and ITS sequences of *F*. *oxysporum*, respectively (Supplementary Table 2). Phylogenetic analysis based on the ITS region sequences of the two isolates (~520–542 bp for both isolates) is shown in Fig. 3c. The results from these analyses confirmed that strain NK-NH1 (KY410238) belongs to the *Fusarium* genus and belongs to the same clade as *F*. *solani*, whereas strain NK-NH2 (KY410239) is related to *F*. *oxysporum*. In addition, two representative sequences of high-throughput sequencing were OTU_1 and OTU_3, which were identified as *F*. *solani* and *F*. *oxysporum* respectively. The two OTUs could match ITS region sequences of the two isolates (Supplementary Fig. 3). Based on phylogenetic relationships, DNA sequencing results, morphological and micro-morphology features, and spore morphology in particular, we deduced that strains NK-NH1 and NK-NH2 were the major *Fusarium* species that exist in the hull surface samples and that NK-NH1 was the dominant fungal community member.

### Degradation of lignin and cellulose by *Fusarium* spp. NK-NH1 and NK-NH2

**Figure 4:**
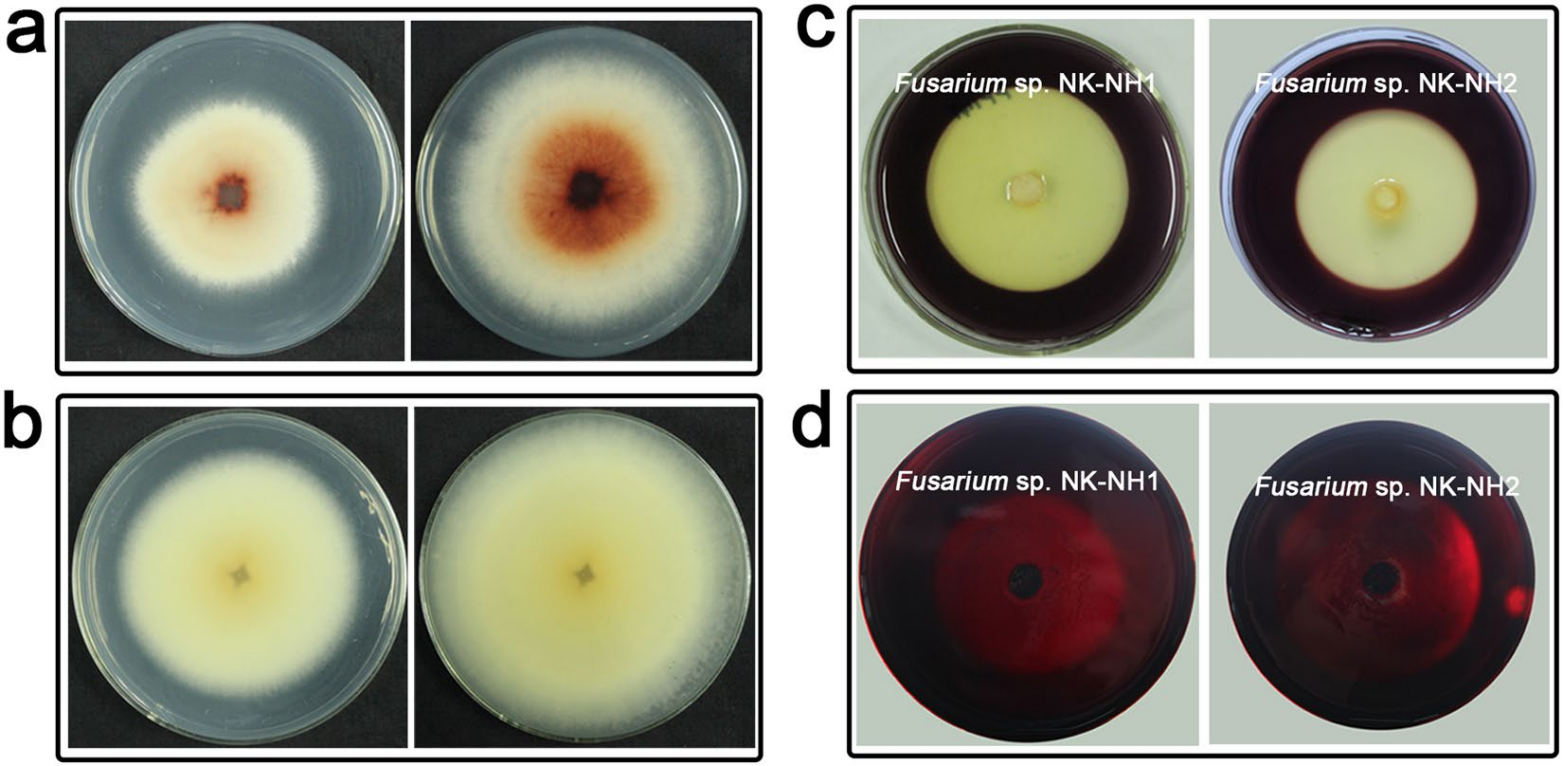
Colony appearances of two isolates on PDA-guaiacol, CMC, and CMC Congo red plates. (**a**) *Fusarium* sp. NK-NH1 grown on PDA-guaiacol plates for 6 (left) and 12 days (right). (**b**) *Fusarium* sp. NK-NH2 grown on PDA-guaiacol plates for 6 (left) and 12 days (right). (**c**) Isolates grown on CMC plates for 4 days. (**d**) Isolates grown on CMC Congo red plates for 6 days.

*Fusarium* sp. NK-NH1 and *Fusarium* sp. NK-NH2 were cultured on PDA plates containing 0.04% (v/v) guaiacol at 28 °C for 6 and 12 days (Fig. 4a–b). *Fusarium* sp. NK-NH1 significantly degraded guaiacol on PDA-guaiacol plates, whereas *Fusarium* sp. NK-NH2 did not exhibit such activity. *Fusarium* sp. NK-NH1 thus exhibited a high capacity for lignin degradation.

We then cultured the isolates on medium with Congo red and carboxymethylcellulose (CMC) to assess whether they could degrade cellulose. The two isolates were cultured on CMC plates for 4 days and CMC Congo red plates for 6 days. After flooding the plates with Gram’s iodine for 5 minutes, both isolate colonies showed clear and distinct zones, indicating that they were both capable of cellulose degradation (Fig. 4c–d).

### Test of biocides to inhibit the growth of *Fusarium* spp. NK-NH1 and NK-NH2

**Figure 5:**
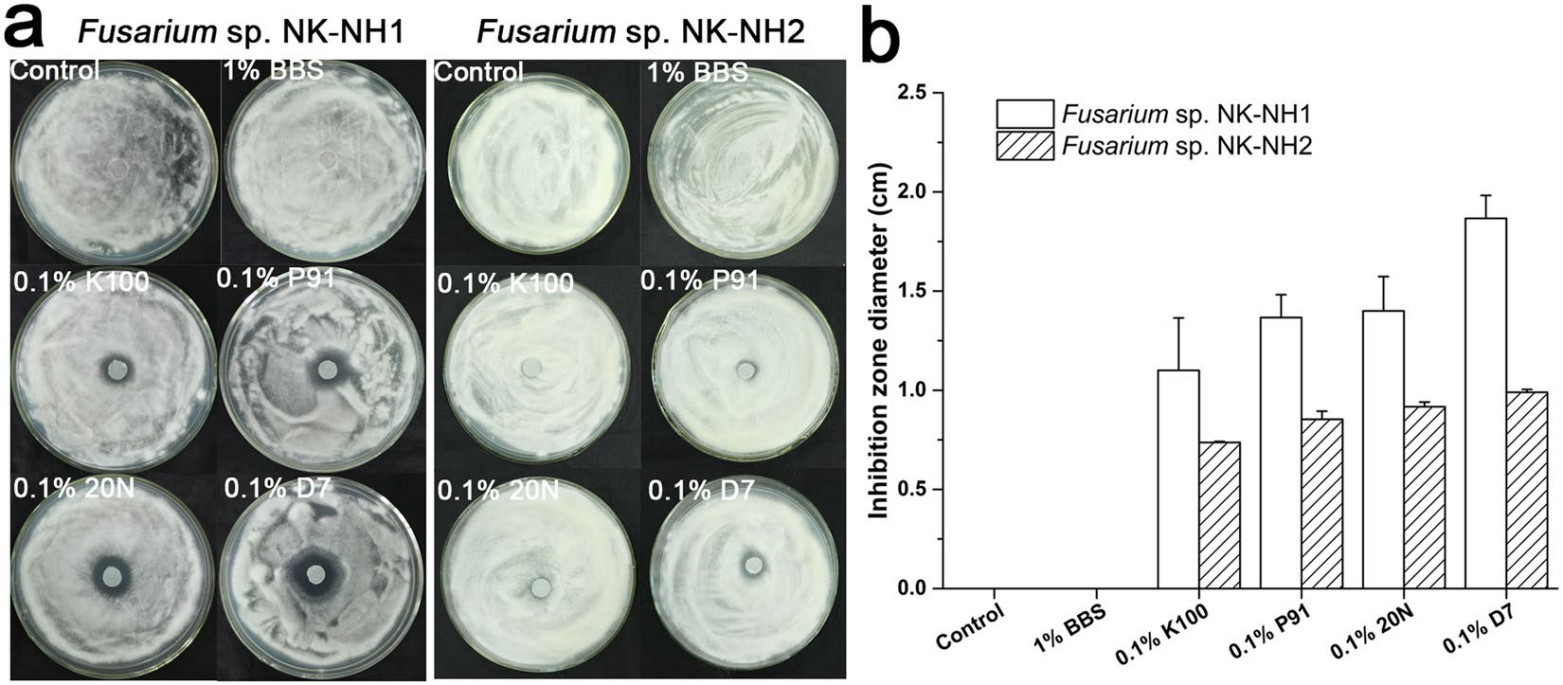
Photograph showing the inhibition of fungal growth by biocides. (**a**) The disks on each PDA plate were loaded with the same concentration of different biocides. Clearing zones indicate where the growth of *Fusarium* spp. NK-NH1 and NH2 was inhibited. (**b**) The inhibition efficiency of different biocides. The ordinate is the diameter of antibiotic inhibition zone. Vertical lines indicate standard deviations of three replicate tests for each.

To determine the efficacy of various antibiotics towards inhibiting *Fusarium* spp. NK-NH1 and NK-NH2 growth, we assayed growth in the presence of five kinds of biocides on PDA media. Agents containing isothiazolinones, such as Preventol^®^ D7, BIT 20N, P91, and Euxyl^®^ K100, suppressed the growth of *Fusarium* spp. NK-NH1 and NK-NH2 at a 0.1% concentration (v/v), as indicated by inhibition rings. However, Borate buffer solution (BBS) at a 1% concentration (w/v) did not suppress the growth of either strain (Fig. 5). Of the two strains, *Fusarium* sp. NK-NH1 was more sensitive to isothiazolinones.

## Discussion

Mitigating the degradation of important cultural artefacts is of critical importance in the maintenance of important cultural heritage items. Here, we analysed the microbial communities present in an excavated 800-year-old shipwreck in order to inform future conservation efforts. Microscopic observations showed that the hull of the Nanhai No. 1 shipwreck suffered from fungal colonization.

High-throughput sequencing of the 16S rRNA V4 regions revealed that the most abundant bacterial phyla of the shipwreck samples were Firmicutes and Proteobacteria, which were found in all of the samples. The Firmicutes genera *Gracilibacillus* and *Alicyclobacillus* were present in all samples and are often associated with earthy and drought environments but have also been detected on salt-degraded monuments^15,16^. *Gracilibacillus* are halotolerant bacteria that may not be able to degrade cellulose but can produce xylanases^17^. Proteobacteria was the other dominant bacterial phylum and was represented by the genera *Marinobacter*, *Halomonas* and *Azoarcus*. *Marinobacter* are frequently identified as hydrocarbon-degrading organisms in a wide variety of marine environments, including lignin-enriched environments^18^. *Halomonas* are halophiles that require high NaCl concentrations for growth. *Halomonas* are highly versatile and can successfully grow in a variety of temperature and pH conditions and, importantly, produce cellulases and xylanases^19,20^. *Azoarcus* are usually found in contaminated water, as they are involved in the degradation of some contaminants, commonly living in soil^21^. Thus its presence on the ship might be associated with the contaminants of sea water. The other bacterial phyla that were found, Bacteroidetes, Actinobacteria, Chloroflexi, Acidobacteria, and Gemmatimonadetes, are all also frequently detected in cultural heritage artefacts and humid environments^22^.

Waterlogged wood from shipwrecks often displays bacterial or soft-rot decay. Cryo-sectioning and scanning electron microscopy indicated that the wood recovered from the *Tektaş Burnu* shipwreck was undergoing extensive degradation caused by erosion bacteria, tunnelling bacteria, and marine borer activity^23^. Wood samples from the rostrum of an excellent workmanship were analysed by 16S rRNA gene-based community sequencing techniques, revealing the presence of *Pseudomonas* spp., *Sphingomonas* spp., *Xanthomonas* spp., *Marinobacter* spp. and *Desulforudis audaxviator*^24^. Microbial decay is also a problem that was encountered in the preservation process of King Henry VIII’s Tudor Warship, the *Mary Rose*. *Alicyclobacillus* and *Acidiphilium* spp. were detected in this particular setting (66% and 34% of the communities, respectively). In addition, clone-library based 16S rRNA gene analyses indicated the presence of Acidobacteria and Bacteroidetes^25^. While samples collected from the Nanhai No. 1, the genera *Idiomarina*, *Aquiflexum*, *Gracilibacillus*, *Bacillus*, *Halomonas*, *Marinobacter*, *Alicyclobacillus and Azoarcus* were the main bacterial taxa. One possible reason for the differences of bacterial community in these cases is the different conditions of preservation. Although the shipwreck was stored in a tank with seawater, the upper deck has been exposed to the air. This brief increase in available oxygen may promote the growth of these dominant bacteria. Soft-rot decay seems to play the largest role in the deterioration of the shipwreck at present. Although a number of fungal genera were detected in wood from the Nanhai No. 1 shipwreck, the *Fusarium* genus was dominant in all samples and accounted for more than 90% of the total fungal communities.

*Fusarium* is not typically known as an important wood-degrading fungal genus but rather a litter- and soil-associated fungus that is important for degrading plant detritus. However, the genus is often isolated from wooden materials and is known to possess high lignocellulolytic activity^26,27^, which was demonstrated by the plate assays in the study. Furthermore, Lavin *et al*. demonstrated that *Fusarium* spp. isolated from document collections were able to form biofilms, produce pigments, and decrease pH, which resulted in structural damage and thus constituted a hazard for document preservation^28^. In this case, the Nanhai No. 1 shipwreck has been waterlogged from approximately 1100 AD until 2007; it is possible that the long time on the sea bottom has changed the structure of the wood so that it is more susceptible to fungal colonization. Moreover, the new conservation conditions, including the increase in oxygen concentration and the reduction in moisture content, will play an overriding role in the colonization of the fungus after the ship is exposed to the museum’s atmospheric environment. Therefore, *Fusarium* spp. should be regarded as a potential threat to the wooden ship. As a follow-up, we will try to prove that *Fusarium* spp. actually degrades waterlogged wood of the Nanhai No. 1 shipwreck under laboratory conditions by measuring the weight loss of waterlogged wood samples or their chemical alteration after inoculation of the fungus.

To properly conserve waterlogged archaeological wood, environmental control is essential because environmental conditions greatly influence microbial growth and their decomposition of wooden cultural properties. Destructive microorganisms are usually active in environments that are wet, oxygenated and warm^29^. Optimal humidity levels for white-rot and brown-rot fungi are in the range of 40%–80%^30^. The waterlogged state of the integral hull could impede microbial activity and confer some protection for the ship. Oxygen concentration is crucial for fungal growth, and the upper deck of Nanhai No. 1 is exposed to air. Due to the large size of the Nanhai No. 1 shipwreck and the intermittent entrance of archaeological workers into the museum to perform archaeological excavation and protection work, maintaining an oxygen-free environment may be problematic, but reducing the oxygen concentration around the ship should be considered. Moreover, the temperature inside the museum is suitable for the growth and survival of most microorganisms and thus likely contributes to the microbial colonization on the ship. The storage of waterlogged excavated wood should be at low temperature (the *Mary Rose* spraying system is approximately 5 °C)^31^.

In addition to controlling the environment as a means to inhibit growth, efficient monitoring and protective measures should be conducted. First, sampling sites should be constantly monitored to assess whether microbial communities are changing, and microbial contamination on shipwreck surfaces should be physically removed on a regular basis. Second, and more importantly, it is necessary to use efficient and low-toxicity biocides to repress the growth of dominant fungi in order to mitigate biodeterioration. Existing commercial agents used to prevent microbial damage to cultural heritage artefacts often involve considerable amounts of potentially hazardous agents and even materials that are poisonous to artefact-protecting personnel and the environment^32^. The high value of cultural heritage protection is such that the risks of any inhibitory agents that are used should be assessed. Biocides that are used should be non-toxic to personnel and to the public and cause no harm to the cultural materials. Although isothiazolinones proved effective to inhibit the activity of *Fusarium* in our laboratory analyses, further testing will be required to ensure that their usage meets the above criteria and will also be compatible with future conservation methods and materials.

Microorganisms clearly play an important role in the biodeterioration of cultural property. However, analysing the microbial communities, as reported here, can be helpful in selecting optimal biocides against microorganisms, thus reducing the biodegradation and destruction on the Nanhai No. 1 shipwreck and other valuable cultural artefacts that are an important part of our historical heritage.

## Methods

### Sample collection

The annual temperature and relative humidity in the Marine Silk Road Museum are 16.4–30.7 °C with an average of 25.6 °C and 63.1–97.2% with an average of 84.1%, respectively. The ship is 30.4 metres long and has a maximum width of approximately 9.8 metres. The types of wood used in the construction of the ship included *Pinus massoniana*, *Fokienia hodginsii*, *Terminalia catappa* (Hainan), *Mischocarpus sundaicus* and *Alnus trabeculosa*.

We sought to investigate the nature of the microbial contamination of the shipwreck at two periods. In April 2015, two samples (NHI.1 and NHI.4) were taken using minimally invasive sampling techniques with sterile scalpels (Supplementary Fig. 2). These samples were collected from areas showing visible mycelia on the outside of the ship’s hull. In October 2015, four samples (NHI.8, NHI.9, NHI.11, and NHI.12) were collected using the methods described above. All samples were placed into 2 mL micro-centrifuge tube and brought to the laboratory in an icebox for subsequent microscopic observation and DNA extraction. The sampling locations on the Nanhai No. 1 ship’s hull are indicated in Fig. 1. Permission to sample was issued by the Marine Silk Road Museum of Guangdong. Yue Chen and Jie Liu from the Chinese Academy of Cultural Heritage (CACH) supervised the sampling process.

### Microscopic observation

Slides were prepared by hand using a razor blade to cut frozen sections, and microscopic observations were then carried out using a transmission light microscope (Leica DM LB2).

### DNA extraction and PCR amplification

Total genomic DNA was extracted from scrapings of the wood surface using the MoBio PowerSoil^®^ DNA Isolation Kit (MO BIO Laboratories, Inc., CA, USA) following the manufacturer’s protocol. Extracted DNA was diluted to 1 ng/μL using sterile water and then stored at −80 °C for subsequent analyses.

Amplification of the bacterial 16S rRNA gene V4 region and the fungal ITS1 region was performed using the universal prokaryotic and fungal primers 515F/806R and the ITS5–1737F/ITS2-2043R (Supplementary Table 3), respectively, with barcodes attached that were unique to each sample^33,34^. All PCR reactions were carried out using Phusion^®^ High-Fidelity PCR Master Mix with GC Buffer (New England Biolabs, UK). Amplifications were carried out in a 50 μL reaction mixture including 25 μL of Master Mix (2X), a 0.5 μM final concentration of the forward and reverse primers, 10 ng of template DNA and nuclease-free water to 50 μL. The PCR conditions were 98 °C for 1 min, followed by 30 cycles of 10 s at 98 °C, 30 s at 50 °C for 16S rRNA gene amplification or 55 °C for ITS region amplification, and 30 s at 72 °C, with a final extension of 5 min at 72 °C.

To visualize PCR amplification success, an equal volume of 1X loading buffer (containing SYBR green) along with PCR products were loaded on a 2% agarose gel. Samples with amplicon bands in the range of 400–450 bp were chosen for further analyses. PCR products from different samples were pooled with equal molar amount. Then, mixture PCR products was purified with Qiagen Gel Extraction Kit (Qiagen, Germany).

### Library preparation and sequencing

The purified amplicons were prepared for Illumina sequencing by constructing a library using the TruSeq^®^ DNA PCR-Free Sample Preparation Kit (Illumina, USA) following the manufacturer’s recommendations. The final library concentrations and quality were checked using a Qubit@ 2.0 Fluorometer (Thermo Scientific) and an Agilent Bioanalyzer 2100 system, respectively. Lastly, the library was sequenced on a Hiseq2500 PE250 platform at the Novogene Bioinformatics Technology Co., Ltd. (Beijing, China), and 250 bp paired-end reads were generated.

### Bioinformatic analyses

Paired-end reads were assigned to samples based on unique barcodes and then trimmed of barcode and primer sequences. Paired-end reads were merged using FLASH (v. 1.2.7)^35^, and the resultant sequences were used as raw tags. Quality filtering of raw tags to obtain high-quality clean tags was performed according to the QIIME (v. 1.7.0)^36^ quality control protocol. Fungal tags were compared with the Unite database (v. 20140703), bacterial tags were compared to the SILVA Gold database (v. 20110519) using the UCHIME algorithm (v. 4.1)^37^ to detect chimaera sequences, and sequences flagged as chimaeras were then removed. The resultant high-quality sequences were used for further analyses. OTU clustering analysis was performed using the Uparse software (v. 7.0.1001)^38^. Sequences with ≥97% similarity in nucleotide identity were assigned to the same OTUs (Operational Taxonomic Units). Representative sequences for each OTU were then used for taxonomic annotation. For each representative fungal sequence, BLAST analysis was performed against the Unite database (v. 20140703)^39^ in QIIME (v. 1.7.0) to taxonomically annotate OTUs. For bacterial OTUs, the Greengenes database (http://greengenes.lbl.gov) was used with the RDP classifier (v. 2.2)^40^ algorithm for taxonomic annotation. Multiple sequence alignments were then conducted using MUSCLE (v. 3.8.31)^41^ in order to compare OTU distributions among samples. Read numbers were normalized to the sample with the least amount of sequences in order to standardize sequence numbers among samples.

The raw sequencing data have been deposited into the NCBI Sequence Read Archive (SRA) under the accession numbers SAMN07311372–SAMN07311377 and SAMN07311378–SAMN07311383 for bacterial and fungal reads, respectively.

### Isolation and identification of dominant fungi

Samples were aseptically inoculated onto potato dextrose agar (PDA) plates directly at the sampling sites and then incubated at 28 °C for 4 days and observed daily. Colonies were transferred to fresh plates to obtain pure isolates. Fungal isolates were identified on the basis of microscopic morphology and gene sequencing.

Pure cultures of isolates were grown on PDA plates for 5 days at 28 °C prior to DNA extraction. DNA was extracted from cultures using the CTAB method^42^. Fungal 28S rRNA genes were amplified using the primers LR0R/LR7^43^. Fungal ITS1-5.8S rRNA-ITS2 genes were amplified with the primers ITS1/ITS4^44^. PCR reaction mixtures consisted of a total volume of 50 μL containing: 1~2 μL of genomic DNA, 5 μL of 10× Reaction Buffer, 4 μL of 2.5 mM dNTP mix, 2 μL of 10 μM ITS1 primer, 2 μL of 10 μM ITS4 primer, 0.5 μL of 5 U/μL Transtaq-T DNA polymerase (TransGen Biotech), and ddH_2_O to 50 μL. PCR products were sequenced by GENEWIZ (Beijing, China), and sequence identities were analysed using the National Center for Biotechnology Information (NCBI) BLAST program (https://blast.ncbi.nlm.nih.gov/Blast.cgi) and the GenBank database. Each isolate was compared against known taxa present in the database. Phylogenetic analyses were conducted using the Molecular Evolutionary Genetics Analysis software (MEGA, v. 6.06) using the neighbour-joining method. Confidence in tree topology was estimated using the bootstrap method (1,000 bootstrap replicates).

### Ligninolytic and cellulolytic enzymatic activity

Lignocellulose degradation by fungi was visualized on plates containing guaiacol, which indicates the activity of lignocellulolytic enzymes that catalyse the oxidative polymerization of guaiacol and result in reddish brown zones in the media^45^. Lignocellulose degradation ability by fungi is directly proportional to the size and depth of the reddish-brown zones. Furthermore, the larger and deeper the reddish-brown zones, the stronger the lignocellulose degrading ability.

Discs of fungi were cut into 0.5 × 0.5 cm sections from the actively growing isolate colony margins and transferred onto PDA plates containing 0.04% (v/v) guaiacol and incubated at 28 °C to assess ligninolytic ability.

Two different media were prepared to assess the ability of isolates to utilize cellulose: (i) CMC agar medium and (ii) CMC Congo red agar medium. CMC agar consisted of 0.2% NaNO_3_, 0.1% K_2_HPO_4_, 0.05% MgSO_4_, 0.05% KCl, 0.2% carboxymethylcellulose (CMC) sodium salt, 0.02% peptone, 1.7% agar and 1 L of tap water. CMC Congo red agar consisted of 0.05% K_2_HPO_4_, 0.025% MgSO_4_, 0.188% carboxymethylcellulose (CMC) sodium salt, 0.02% Congo red, 0.2% gelatin, 1.7% agar and 1 L of tap water^46^.Gram’s iodine consisted of 2.0 g of KI and 1.0 g of iodine dissolved in 300 mL of distilled water. Discs of fungi were cut with a 7.5 mm diameter from the actively growing colony margins of isolates. The discs were then transferred onto CMC and CMC Congo red agar plates and incubated at 28 °C for 4 and 6 days, respectively. After incubation, 5 mL of Gram’s iodine was added to CMC and CMC Congo red agar plates, and the plates were incubated at room temperature in the dark for 5 minutes. All media were autoclaved for 20 minutes at 121 °C.

### Biocide inhibition effectiveness

The application of biocides is one of the most important means to control microbial deterioration. Commercial agents such as Biotin^®^ T and Preventol^®^ RI 80 (isothiazolinones) have been widely applied on inorganic substrates that comprise cultural heritage materials, such as stone and mortars^47,48^. The active agent, isothiazolinones, is considered to be not only effective but also preventive when applied to paper and stone materials^49^. Borate buffer solution (BBS) has also been used to mitigate microbial deterioration following the full-scale excavation of the shipwreck that began in 2013. In addition to these biocide agents, we selected four additional agents, whose main component is isothiazolinones, in order to test their biocidal efficacy in the laboratory (Supplementary Table 4).

Discs of fungi were inoculated onto PDA plates and incubated at 28 °C for the preparation of a spore suspension. After five days’ incubation, 10 mL of 0.1% tween−80 was added to each PDA plate, and conidia were scraped by a glass spreading rod and transferred into a 50 mL concentrator bowl. The spore suspension was filtered with three layers of sterile gauze, followed by centrifugation at 4,500 rpm for 10 minutes. The supernatant was discarded, and pellets were re-dissolved in sterile water. Then, 2.4 × 10^11^ conidia from a spore suspension of each strain were then inoculated on to PDA plates and were dispersed with a glass spreading rod. Filter paper was cut to make a circle of 0.7 cm diameter, loaded with 30 μL of different biocides and placed in the centre of PDA plates. The plates were examined for clear zones after incubation at 28 °C for 3 days. The presence of any clear zone that formed around the filter paper was recorded as an indication of inhibition against the fungal species. Each antimicrobial agent test comprised three replicates, and sterile water was used as a control.

### Data availability statement

The datasets supporting the conclusions of this study are included within this manuscript and its supplementary files.

## Electronic supplementary material

supplementary Information
