# Supplementary material for: Microbial community analysis and biodeterioration of waterlogged archaeological wood from the Nanhai No. 1 shipwreck during storage
Source: Sci Rep. 2018 May 8;8:7170. doi: 10.1038/s41598-018-25484-8 (PMC5940862; doi:10.1038/s41598-018-25484-8)
Supplement: Supplementary file 1 — supplementary Information [file 41598_2018_25484_MOESM1_ESM.pdf]

Microbial community analysis and biodeterioration of waterlogged archaeological wood from the Nanhai No. 1 shipwreck during storage

Zijun Liu[1], Tongtong Fu[1], Cuiting Hu[1], Dawa Shen[2], Nicola Macchioni[3], Lorena Sozzi[3], Yue Chen[2], Jie Liu[2], Xingling Tian[2], Qinya Ge[2], Zhengteng Feng[4], Huiru Liu[4], Zhiguo Zhang[5]*, Jiao Pan[1]*

1) Ministry of Education Key Laboratory of Molecular Microbiology and Technology, Department of Microbiology, College of Life Sciences, Nankai University, Tianjin 300071, P.R. China.
2) Chinese Academy of Cultural Heritage, Beijing 100029, P.R. China.
3) CNR-IVALSA, Via Madonna del Piano 10, 1-50019 Sesto Fiorentino, Italy.
4) Maritime Silk Road Museum, Yangjiang, Guangdong province 529500, P.R. China.
5) National Center of Underwater Cultural Heritage, Beijing 100192, P.R. China.

*Corresponding author:
Dr. Zhiguo Zhang, email: zzgwys@126.com
Dr. Jiao Pan, email: panjiaonk@nankai.edu.cn

Supplementary Table 1. Relative abundances of dominant bacteria among samples. (%).

Bacterial phylum/genus	Taxonomy	NHI.1	NHI.4	NHI.8	NHI.9	NHI.11	NHI.12
Phylum level	Firmicutes	51.95	6.3	81.92	56.34	38.1	21.33
	Proteobacteria	33.6	76.87	11.33	31.43	40.71	34.04
	Bacteroidetes	4.31	5.71	3.6	7.3	4.47	38.09
	Actinobacteria	1.94	2.47	1.54	3.01	11.58	4.77
	Chloroflexi	2.66	2.17	0.13	0.17	0.88	0.25
	Acidobacteria	1.32	1.69	0.1	0.13	2.13	0.02
	Deinococcus-Thermus	0.27	0.26	0.15	1.29	0.58	1.09
	Gemmatimonadetes	0.84	1.16	0.04	0.09	0.44	0.01
	Chlorobi	0.78	0.63	0.01	0	0	0
	Planctomycetes	0.59	0.57	0	0	0.04	0.01
	Others	1.74	2.17	1.18	0.23	1.07	0.38
Genus level	*Idiomarina*	0.3	34.43	0.3	1.92	0.77	1.74
	Aquiflexum	0	0	0.96	0.4	1.77	31.39
	Gracilibacillus	1.86	0.6	30.15	4.4	4.42	3.6
	Bacillus	26.53	3.43	10.51	3.66	3.37	1.04
	Halomonas	14.15	12.12	2.83	13.03	4.18	5.77
	unidentified_Mitochondria	0.06	0.04	1.19	0.3	12.93	0.59
	Marinobacter	0.09	0.05	1.1	1.14	5.58	11.77
	Alicyclobacillus	0.77	0.25	0.68	3.79	6.3	0.32
	Azoarcus	2.06	1.22	0.68	0.45	1.49	4.75
	Psychrobacter	0.01	3.38	0	0	0	0
	Others	54.16	44.5	51.6	70.91	59.19	39.03

Supplementary Table 2. Gene-based identification results for strains NK-NH1 and NK-NH2

Strain name	Gene sequence	Closest match	BLAST Identity	GenBank accession
NK-NH1	28S rRNA gene	*F. solani*	99%	FJ345352
NK-NH1	ITS	*F. solani*	100%	KT87641
NK-NH2	28S rRNA gene	*F. oxysporum*	100%	EF590327
NK-NH2	ITS	*F. oxysporum*	99%	JN903939

Supplementary Table 3. Primer sequences used in this study.

Primer pair	Targeting region	Sequence (5`-3`)	Reference
515F/806R	16S rRNA V4	Forward: GTGCCAGCMGCCGCGGTAA<br>Reverse: GGACTACHVGGGTWTCTAAT	33
ITS5-1737F/<br>ITS2-2043R	ITS 1	Forward: GGAAGTAAAAGTCGTAACAAGG<br>Reverse: GCTGCGTTCTTCATCGATGC	34
LR0R/LR7	28S rRNA	Forward: ACCCGCTGAACTTAAGC<br>Reverse: TACTACCACCAAGATCT	43
ITS1/ITS4	ITS1, ITS2	Forward: TCCGTAGGTGAACCTGCGG<br>Reverse: CCTCCGCTTATTGATATGC	44

Supplementary Table 4. Biocides used in this study.

Antimicrobial agent	Main components	Manufacturers
1 % Borate buffer solution (BBS)	0.7 % (w/v) Boric acid and 0.3 % (w/v) Borax	Genview, USA
Euxyl® K100	Liquid preparation based on Methylchloroisothiazolinone, Methylisothiazolinone and Benzyl Alcohol	Schülke, Germany
Preventol® P91	Aqueous preparation of min. 9.0 % 2-bromo-2-nitropropane-1,3-diol (Bronopol) and min. 1.0 % isothiazolinones.	Lanxess, Germany
Preventol® BIT 20N	~20 % aqueous-glycolic solution of 1,2-Benzisothiazolin-3-one (BIT).	Lanxess, Germany
Preventol® D7	Aqueous formulation of isothiazolinones	Lanxess, Germany

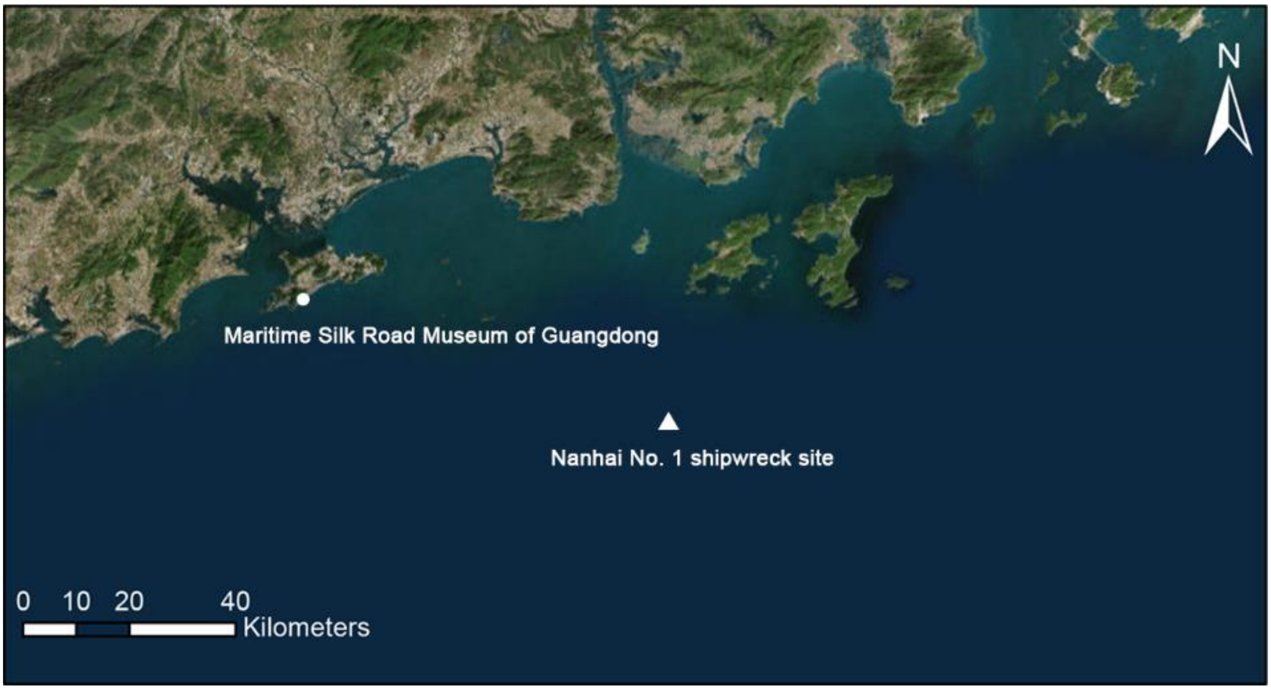

Supplementary Figure 1. Location of the Nanhai No. 1 shipwreck site. The map was drawn by ArcGIS v. 10.2 (www.esri.com/arcgis).

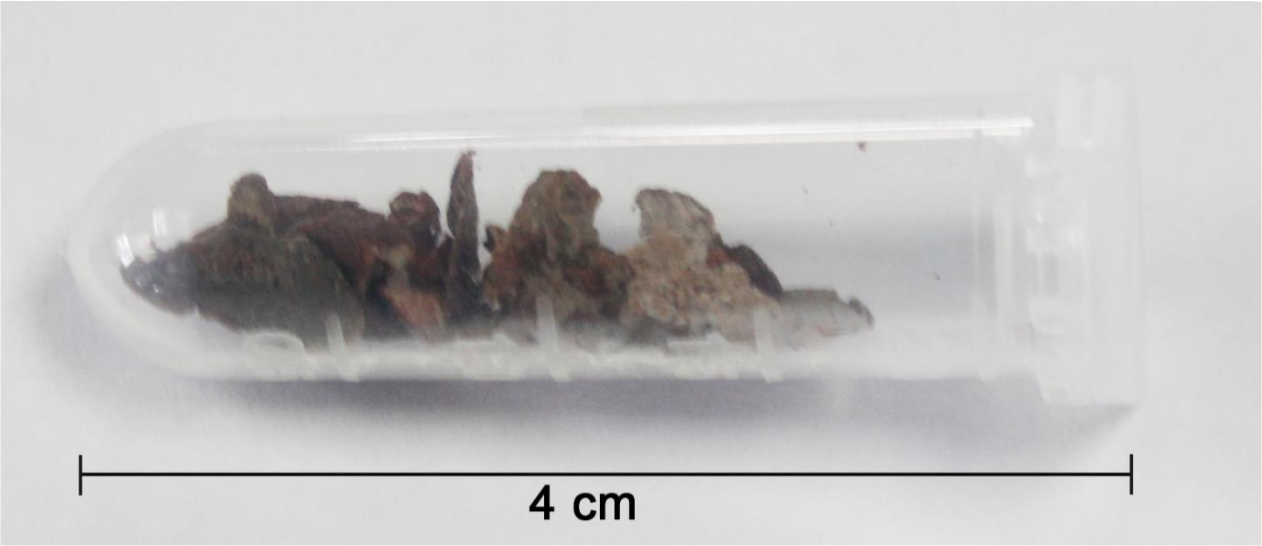

Supplementary Figure 2. Wood scrapes taken from Nanhai No.1.

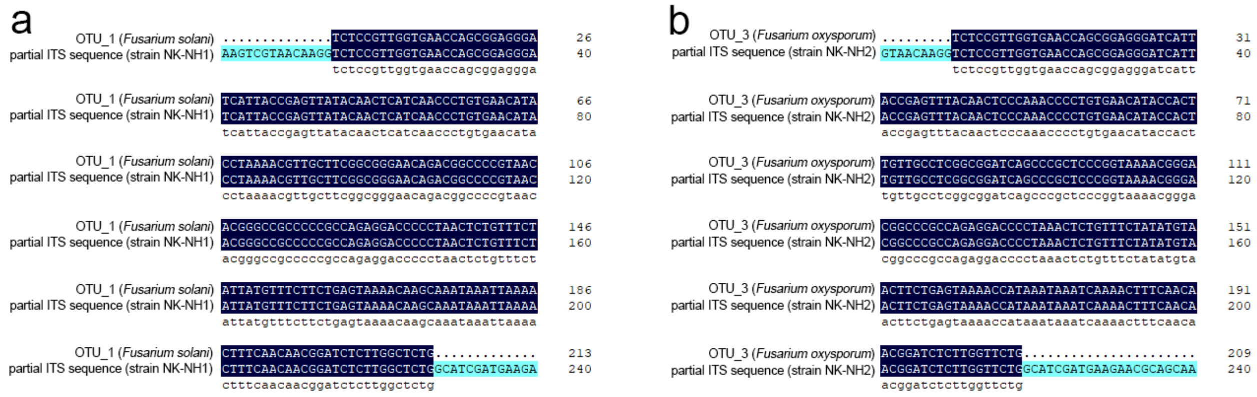

Supplementary Figure 3. (a) OTU_1 and partial ITS sequence of strain NK-NH1. (a) OTU_3 and partial ITS sequence of strain NK-NH2. The alignment was carried out by DNAMAN (v. 8.0)
